# Signals of Ezh2, Src, and Akt Involve in Myostatin-Pax7 Pathways Regulating the Myogenic Fate Determination during the Sheep Myoblast Proliferation and Differentiation

**DOI:** 10.1371/journal.pone.0120956

**Published:** 2015-03-26

**Authors:** Caihong Wei, Hangxing Ren, Lingyang Xu, Li Li, Ruizao Liu, Li Zhang, Fuping Zhao, Jian Lu, Xiaoning Zhang, Lixin Du

**Affiliations:** 1 National Center for Molecular Genetics and Breeding of Animal, Institute of Animal Sciences, Chinese Academy of Agricultural Sciences, Beijing, China; 2 Chongqing Academy of Animal Sciences, Rongchang, Chongqing, China; 3 Institute of Animal Genetics and Breeding, College of Animal Science and Technology, Sichuan Agricultural University, Chengdu, China; Stem Cell Research Institute, BELGIUM

## Abstract

Myostatin and Pax7 have been well documented individually, however, the mechanism by which Myostatin regulates Pax7 is seldom reported. Here, based on muscle transcriptome analysis in Texel (*Myostatin* mutant) and Ujumqin (wild type) sheep across the five fetal stages, we constructed and examined the Myostatin-Pax7 pathways in muscle. Then we validated the signals by RNAi in the proliferating and differentiating sheep myoblasts in vitro at mRNA, protein, and cell morphological levels. We reveal that Myostatin signals to Pax7 at least through Ezh2, Src, and Akt during the sheep myoblast proliferation and differentiation. Other signals such as p38MAPK, mTOR, Erk1/2, Wnt, Bmp2, Smad, Tgfb1, and p21 are most probably involved in the Myostatin-affected myogenic events. Myostatin knockdown significantly reduces the counts of nucleus and myotube, but not the fusion index of myoblasts during cell differentiation. In addition, findings also indicate that Myostatin is required for normal myogenic differentiation of the sheep myoblasts, which is different from the C2C12 myoblasts. We expand the regulatory network of Myostatin-Pax7 pathways and first illustrate that Myostatin as a global regulator participates in the epigenetic events involved in myogenesis, which contributes to understand the molecular mechanism of Myostatin in regulation of myogenesis.

## Introduction

Myostatin, a member of the transforming growth factor-β (TGF-β) family, is predominantly expressed and secreted by skeletal muscle and functions as a negative regulator of muscle growth. Mutations in *Myostatin* gene lead to a hypertrophic phenotype in mice, sheep, cattle, dog, and human [1–6], suggesting that this growth factor alters the myogenic signals during the prenatal development. Surely, we recently demonstrate that there are crucial molecular events involved in the large-scale myoblast fusion in fetal sheep [7]. On the other hand, an important myogenic regulatory factor, the paired box transcription factor Pax7 plays an essential role for early and postnatal myogenesis [8–11]. Pax7 has been found to play a crucial role in myogenic cell fate determination [9, 11–13] and myogenic cell behavior [10, 14–21]. Our recent findings demonstrate that there are more Pax7-positive cells (satellite cells or fetal myoblasts) in the fetal muscle of Texel sheep (a natural mutation in *Myostatin*) than in Ujumqin sheep (wild type) [22], suggesting that *Myostatin* mutation changes Pax7 expression in muscle during fetus development. In myogenesis, Myostatin signaling through Smad2, Smad3 and Smad4 is regulated by the inhibitory Smad7 via a negative feedback mechanism [23, 24], and the p38 MAPK played an important role in regulation of GDF-8-activated Smad pathways [25]. Myostatin-stimulated activation of Erk1/2 negatively regulates myogenic differentiation [26]. In vivo, Myostatin suppresses heart and skeletal muscle growth by negatively regulating Akt and mTOR related signals [27, 28]. In addition, Wnt/beta–catenin, PI3K, NF-kappaB, and Hippo are involved in Myostatin signaling as well [29–33]. However, there are few pathways reported through which Myostatin regulates Pax7 in myogenesis. Up to date, only the Erk1/2 signal is found to be associated with Myostatin-modulated Pax7 expression in muscle of adult mice [34]. It is unclear whether there are other signals associated with Myostatin-modulated Pax7 expression except Erk1/2 in muscle cells. To elucidate comprehensively the mechanism by which Myostatin affects Pax7 expression to orchestrate the myogenic process, in this article, based on fetal muscle microarray analysis in Texel and Ujumqin sheep, we constructed the Myostatin-Pax7 pathways by the IPA program, and further swept and validated the potential signals involved in the sheep myoblasts comprehensively. We demonstrate that Myostatin differentially signals to Pax7 through multiple signal pathways to affect the sheep myoblast proliferation and differentiation. Our findings expand the network of Myostatin-Pax7 pathways and first suggest that Myostatin as a global regulator may participate in the epigenetic event mediated by Ezh2 and Src for decision of muscle cell fate.

## Materials and Methods

### Animals

In the present study, the Texel is a *Myostatin* mutant while the Ujumqin serves as a wild type control [35]. Texel and Ujumqin sheep were obtained from a sheep stud farm located in Youyu, Shan Xi Province. Three pregnant ewes from each breed were subject to caesarean section to collect the fetuses at 70, 85, 100, 120, and 135 d of gestation, and then the longissimus dorsi (LD) muscle was isolated from the individual fetus at each developmental stage as previously described [22]. All experimental and surgical procedures were approved by the Biological Studies Animal Care and Use Committee, Shanxi Province, Peoples Republic of China. Samples were then processed for gene expression analysis as described below.

### Microarray analysis

The sheep-specific transcriptome-wide microarray (Agilent) contained three samples at least at each development stage for each breed. Microarray analysis was performed as we previously described [22]. The original microarray data deposited in the NCBI database (GSE23563), part of which have previously already been published as a breed comparison at the same developmental stage [22]. In the present study, we try to define the Myostatin-Pax7 pathways using two differential gene lists from each breed. To identify differentially expressed (DE) genes, one-way ANOVA was applied to two within-breed contrasts throughout the five developmental stages and the corrected p-value < 0.05 indicates significant difference.

### Platform for building pathways

The Ingenuity Pathways Analysis (IPA) program (http://www.ingenuity.com) was used to construct the Myostatin-Pax7 pathways. Data for analysis were two differentially expressed gene lists for Texel and Ujumqin sheep across five fetal stages, respectively (S1 and S2 Tables). Significant differences between groups were further compared using Fisher’s exact test, and p< 0.05 were considered significant.

### Cell culture, transfection, proliferation and differentiation

The sheep myoblasts were isolated from the longissimus muscle of adult female Ujumqin sheep in our lab [36], and cells were seeded on 24-well plate at a density of 200,000 cells/ml. The lentiviral vector expressing sheep *Myostatin* shRNAs was generated as recently described by our lab [37]. For knockdown of *Myostatin* in myoblasts, cells were infected with 50 of multiplicity of infection (MOI) with a final concentration of 4 μg/μL polybrene, 1mL Opti-MEM was added in 1.5mL centrifugal tube, with addition of corresponding volume of lentivirus and polybrene. The cells were then incubated at 37°C and 5% carbon dioxide (CO2) for 12 h.

The MTT method was used for proliferation assay. Briefly, the sheep myoblasts were seeded in 96-well plate (3,000 cells/well) and maintained in growth medium (GM) (DME containing 10% FBS) at 37°C and 5% CO2 for 7 days. Cells were incubated for 1 h with 10 μM BrdU for examination of the absorbance at 1, 3, 5, and 7 days by a spectrophotometer. The amount of the absorbance of Brdu is proportional to the proliferation rate of cells. The RNA and protein levels of signal molecules in proliferating cells were detected at 3d by quantitative real-time PCR and Western blot. For differentiation assay, the shRNA-infected and the control myoblasts were cultured in differentiation medium (DM) (DME containing 2% horse serum) at 37°C and 5% CO2 for 7 days. Cells were collected at day 3 for examination of mRNA and protein expression, as well as differentiation by the nuclei DAPI staining. Fusion Index (FI) was calculated for evaluating the myogenic potential of myoblasts at this stage.

### Transfection of siRNA

The sheep primary myoblasts were isolated from the muscle of newborn lamb as described [38]. Cells were seeded (2×10^5^ cells/well) in 6-well plates. After 24 h of incubation, they were transfected with *Akt*-siRNA, *Ezh2*-siRNA, and *Src*-siRNA, in serum-free medium using Lipofectamine 2000 (Invitrogen) respectively. Vector (10 μg) and 20 μl Lipofectamine 2000 were mixed and incubated for 15 min at room temperature. The solution was then added to the primary myoblasts and incubated without serum or antibiotics for 6 h, after which the medium was replaced with medium containing serum, but no antibiotics.

### Quantitative real-time PCR (qPCR) analysis

Total RNA was used to make cDNA using the PrimeScript 1st Strand cDNA Synthesis Kit (Takara). qPCR was performed on an ABI 7500 instrument using the Fast EvaGreen Master Mix (Biotium). Thermal cycling consisted of an initial step at 95°C for 10 min followed by 40 cycles at 95°C for 30s and 62°C for 30s. The qPCR measurements were performed in triplicate for each cDNA sample and gene expression was quantified relative to either *RPS18*, *ACTB*, or *GAPDH* expression using the 2-ΔCt method. Data were analyzed using SAS 8.0.

### Western blotting

Equal amounts of proteins from the cell extracts were used for Western blotting. Briefly, 20 μg of protein per sample was subjected to electrophoresis on 12% sodium dodecyl sulfate polyacrylamide gel electrophoresis (SDS-PAGE) gels. The separated proteins were transferred onto nitrocellulose membranes, which were incubated overnight with one of the 20 primary antibodies (S1 File): anti-ERK1 (pY204) + ERK2 (pY187); anti-CyclinE; anti-Src (phospho Y529); anti-Src; anti-Ezh2 (phospho T487); anti- Ezh2; anti-Akt (phospho T308); anti-Cdk2 (phospho T39); anti-p21 (phospho S146); anti-Smad3 (phospho S423 + S425); anti-Histone H3 (tri methyl K4); anti-Histone H3 (phospho S10); anti-Bmp2; anti-mTOR (phospho S2448); anti- Myostatin; anti-Phospho-p38 MAPK (Thr180/Tyr182); anti-Phospho-Rb (Ser807/811); anti-Bmi1; anti-Jnk1 (phospho T183 + Y185). The membranes were washed (5×3min) with TBST and then incubated with secondary antibodies [goat anti-rabbit IgG (H+L), HRP (Jackson), 1:4000] for 40 minutes at room temperature. The membranes were washed above, and HRP activity was visualized using the Fusion X7 imaging system with the Bio1D software (Vuilbert-Lourmat).

## Results

### Differential Myostatin-Pax7 pathways in muscle between breeds

**Fig 1:**
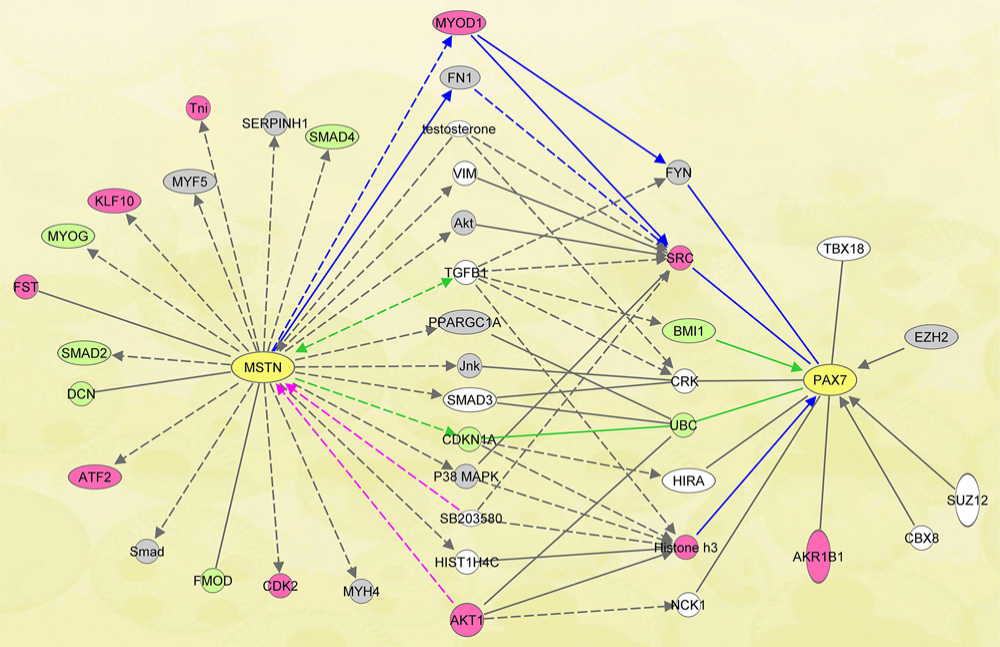
Myostatin-Pax7 pathways in muscle in Texel and Ujumqin sheep. The shortest Myostatin-Pax7 pathways in Texel and Ujumqin were generated using the tools in IPA. The gray ones indicate the DE genes in muscle for both breeds during the five fetal stages. The pink ones indicate the genes differentially expressed in Texel, and the pale green in Ujumqin. The physically direct or indirect interaction between genes is indicated as the solid line or dotted line in the figure.

For within breeds across developmental time, we identified 3711 and 3521 differentially expressed (DE) probes in Texel and Ujumqin sheep, respectively. With annotation by BLASTN, these probes matched 2807 and 2251 human genes, respectively (S1 and S2 Tables). Then we built the Myostatin-Pax7 pathways (Fig. 1) by IPA using the two annotated gene lists.

According to the characteristics of gene expression in each breed, there were three classes of DE genes in the pathways (Fig. 1). Class I, those differentially expressed only in Texel sheep including *Tni*, *Klf10*, *Fst*, *Atf2*, *Cdk2*, *Myod1*, *Src*, *Histone h3*, and *Akr1b1*. Class II, those uniquely differentially expressed in Ujumqin sheep such as *Smad4*, *Myod*, *Smad2*, *Dcn*, *Fmod*, *Cdkn1a*, *Ubc*, and *Bmi1*. Class III, those DE genes shared in both breeds of sheep (named as “co-expressed” genes), such as *Serpinh1*, *Myf5*, *Smad*, *Myh4*, *Fn1*, *Akt*, *Pparg1a*, *Jnk*, *p38MAPK*, *Fyn* and *Ezh2*. Accordingly, those pathways containing the exclusive DE genes in either breed may be involved in breed-specific regulation of muscle development. Whereas the pathways containing the “co-expressed” genes mean that, whether there is mutation in *Myostatin* or not, these pathways are doomed to be differentially expressed and basically indispensable for Myostatin-regulated Pax7 expression in both breeds.

**Fig 2:**
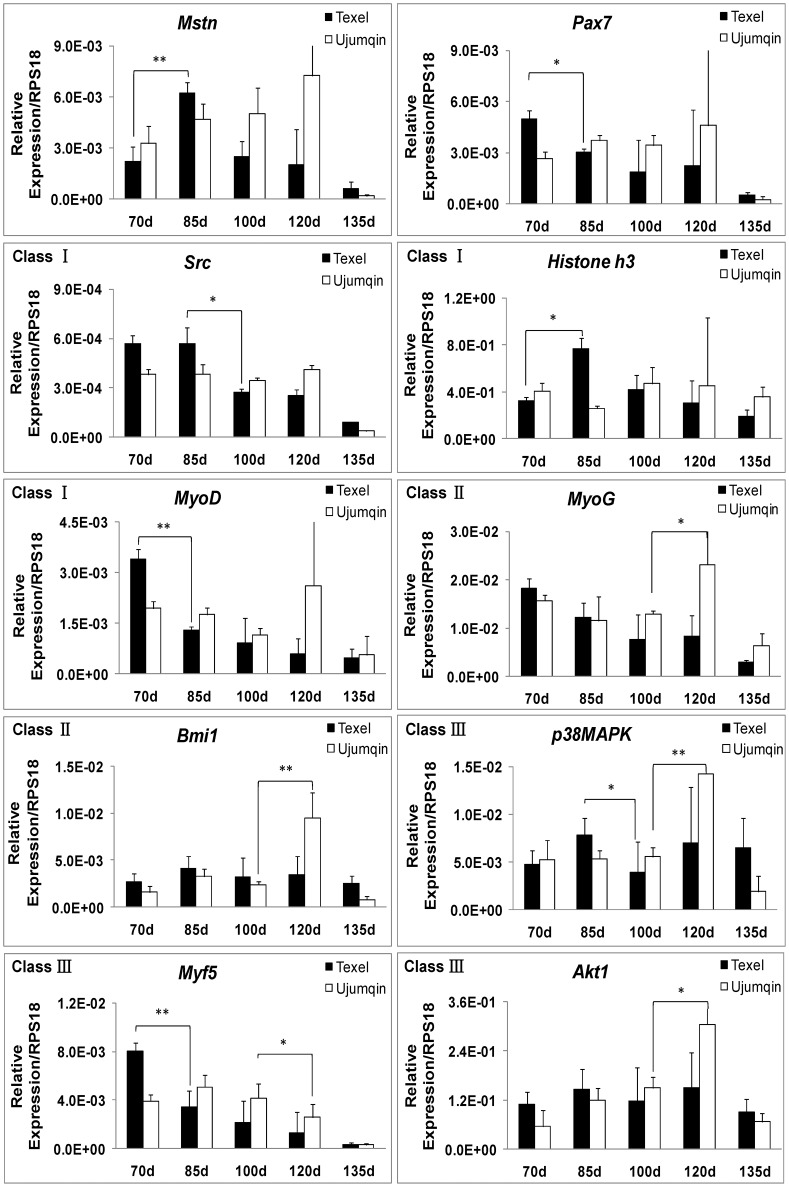
Differential gene expression in muscle in two breeds of sheep by qPCR. The DE genes from microarray analysis were classified into three groups according to the breed-specific characteristic. The Class I are Texel-specific, and the Class II are Ujumqin-specific DE genes. The Class III are those genes differentially expressed in both breeds across the fetal period examined. Gene expression was quantified relative to *RPS18* expression using the 2-△Ct method. The expression data in either breed are shown as the mean + SD (n = 3). * P < 0.05, ** P < 0.01.

To confirm the pathways involved, we examined some of DE genes including *Akt*, *p38MAPK*, *Myf5*, *Bmi1*, *Src*, *Histone h3*, *Myod*, *Myog*, *Mstn*, and *Pax7* in muscle in Fig. 1 by qPCR. As indicated in microarray analysis, the breed-specific DE genes were well classified (Fig. 2), which indicates that these genes may be associated with the regulation of Myostatin-Pax7 pathways. Since the myogenic regulatory factors Myf5, Myod, and Myog are downstream genes of Pax7 [13, 39], they were excluded from the present pathway analysis. Clearly, there is a negative correlation between the mRNA level of *Myostatin* and *Pax7* in muscle at earlier stages (70d and 85d) (Fig. 2). We consider that, the differences of Myostatin-Pax7 pathways probably play key roles in the postnatal muscularity between Texel and Ujumqin sheep.

### Ezh2, Akt, mTOR, p38MAPK, and Erk1/2 involve in promotion of myoblast proliferation by Myostatin knockdown

**Fig 3:**
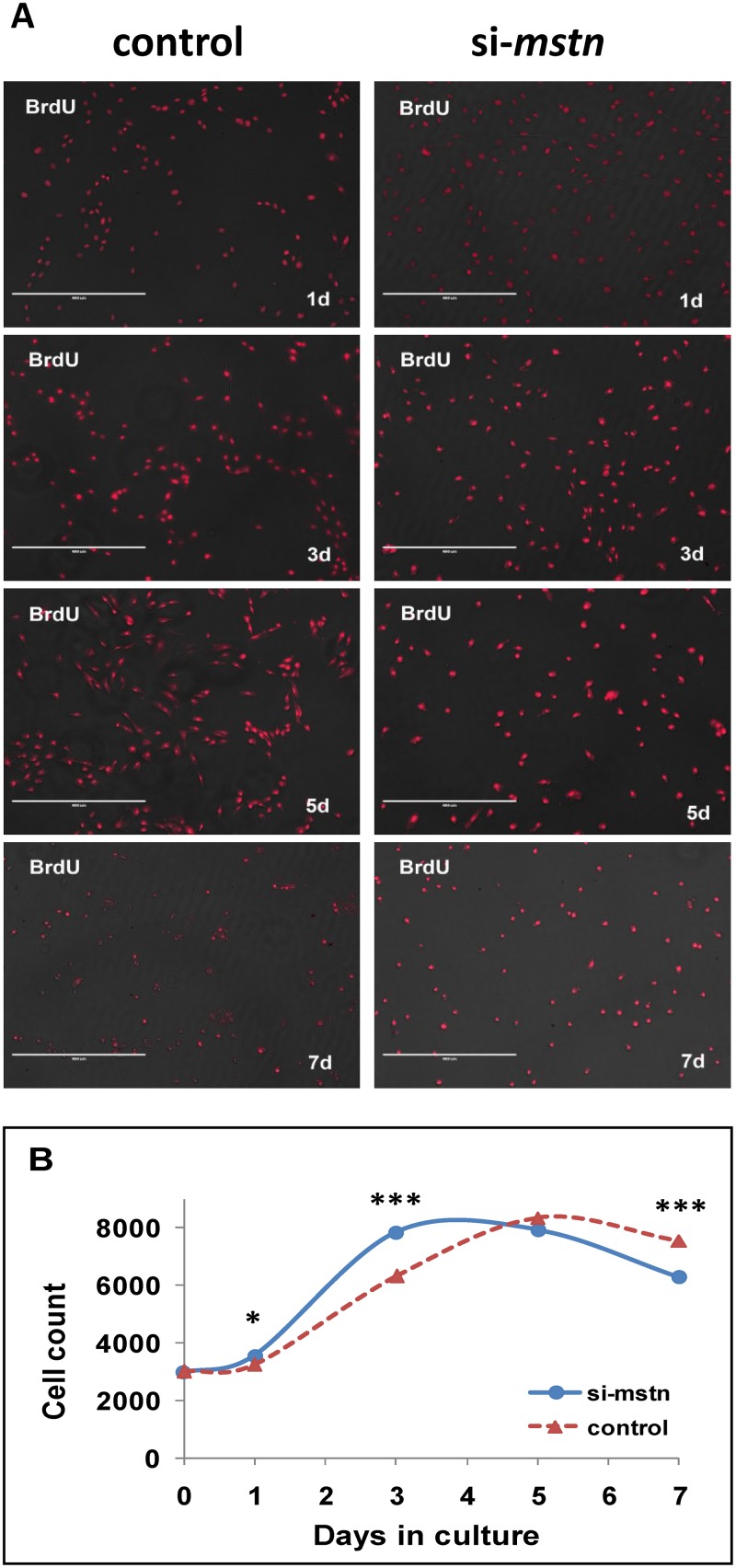
*Mstn* knockdown stimulates the sheep myoblast proliferation. Sheep myoblasts, infected by the lentiviral vector expressing sheep *Myostatin* shRNAs and the negative control vector respectively, were harvested in GM (DME containing 10% FBS) at 1, 3, 5, and 7 days for examination of cell proliferation. (A): the proliferating myoblasts marked with BrdU. (B): variations of cell count measured by the MTT method. * P < 0.05, *** P < 0.001.

**Fig 4:**
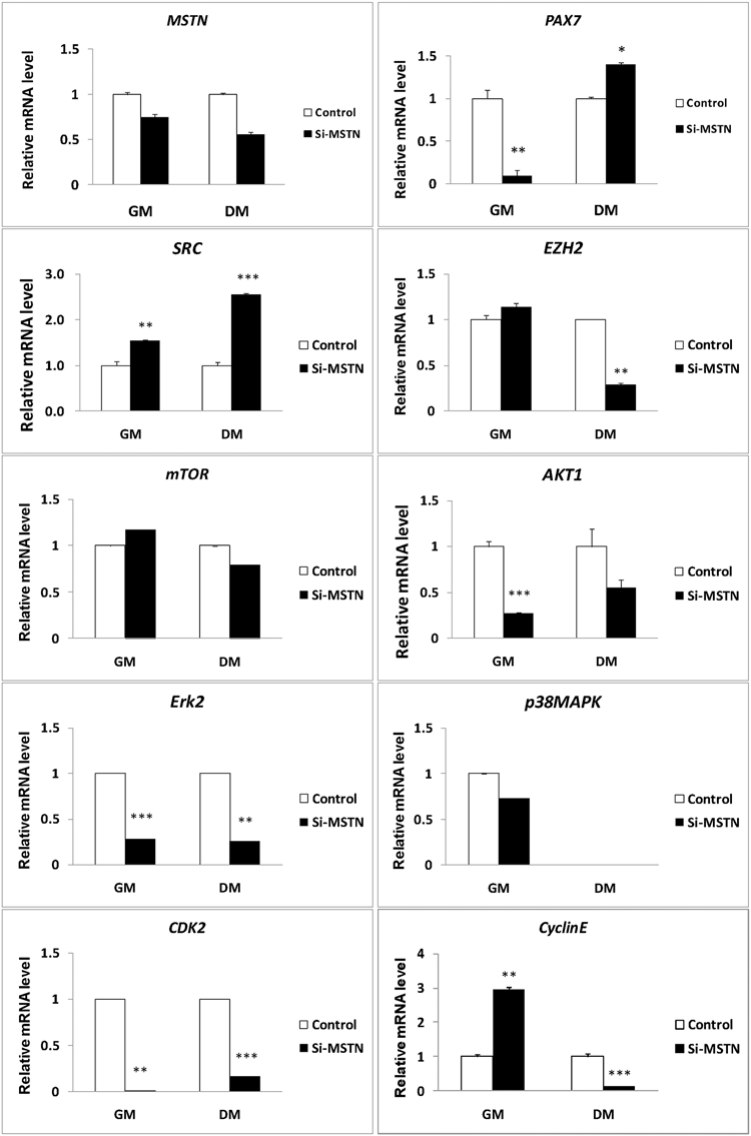
Effect of *Mstn* knockdown on the signals in the sheep myoblasts at the mRNA level. Sheep myoblasts infected by the *Myostatin* shRNAs (si-*mstn*) or control (ctrl) vectors were cultured in either GM or DM for 3 days. Cell lysates were used for gene expression by qPCR. Gene expression was quantified relative to *ACTB* expression using the 2-△△Ct method. The data are shown as the mean + SD (n = 3). * P < 0.05, ** P < 0.01, *** P < 0.001.

**Fig 5:**
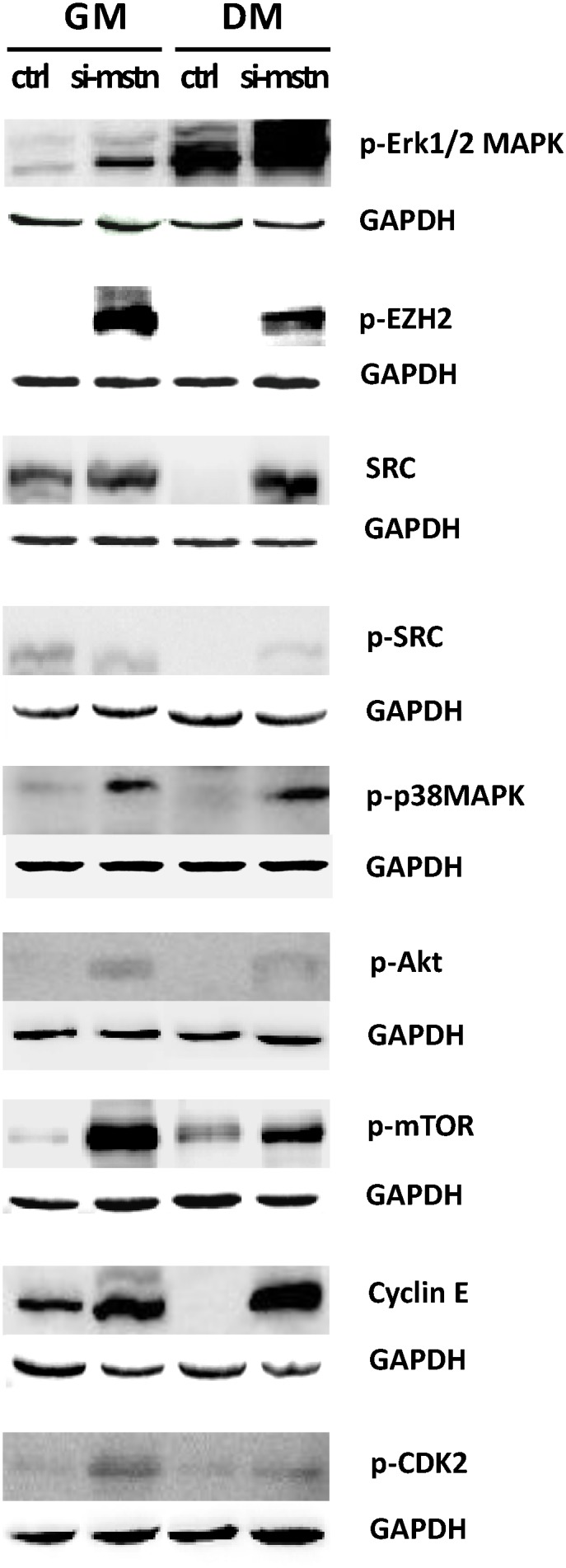
Effect of *Mstn* knockdown on the signals in the sheep myoblasts at the protein level. Sheep myoblasts infected by the *Myostatin* shRNAs (si-*mstn*) or control (ctrl) vectors were cultured in either GM or DM for 3 days. Cell lysates were immunoblotted with p-p38MAPK, p-Erk1/2, Src, p-Src, p-Ezh2, p-mTOR, p-Akt, p-Cdk2, and CyclinE antibodies as described in Materials and Methods. Equal loading was monitored by blotting the membranes with antibodies against those protein above and GAPDH, respectively.

**Fig 6:**
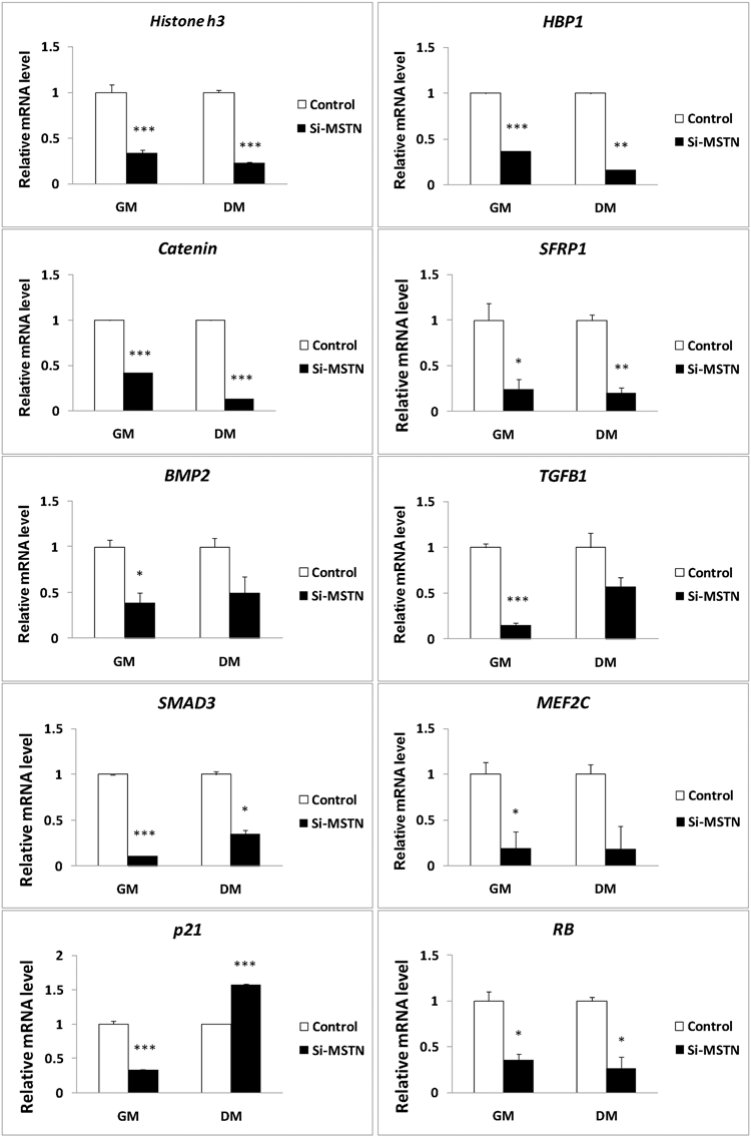
*Mstn* knockdown changes the transcription of other signals in the sheep myoblasts. Gene expression was examined in the infected myoblasts (si-*mstn*) and the negative control (ctrl) under GM or DM by qPCR, respectively. Expression was quantified relative to *ACTB* expression in each treatment using the 2-△△Ct method. The data are shown as the mean + SD (n = 3). * P < 0.05, ** P < 0.01, *** P < 0.001.

To further validate the potential signals above derived from the muscle transcriptome analyses, we constructed the lentiviral siRNA vector for *Myostatin* [37] and examined their expression in the proliferating sheep myoblasts. We demonstrated that *Myostatin* knockdown promoted the myoblast proliferation at early stage in GM (Fig. 3). On the other hand, inhibition of *Myostatin* significantly decreased the mRNA levels of *Pax7*, *Akt1*, *Erk2*, and *Cdk2*, but increased the mRNAs levels of *Src* and *CyclinE* in the proliferating sheep myoblasts significantly (Fig. 4). With the continuation of culture in GM, the cell counts were significantly decreased due to apoptosis as compared with the control at 5 days. Western blot analysis showed that expression of p-Ezh2, p-mTOR, p-Akt, p-Erk1/2, p-p38MAPK, Cdk2, and CyclinE were significantly higher in the siRNA-*Myostatin* infected myoblasts than those in the control (Fig. 5). Higher levels of Cdk2 and CyclinE indicated that more infected myoblasts did not exit the cell cycle, which contributed to the myoblast proliferation at this time point. Although expression of Catenin, Sfrp1, Hbp1, Bmp2, Histone h3, Smad3, Tgfb1, Mef2c, p21and Rb were not detectable by Western blot (data not shown), we can not easily exclude them from the *Myostatin*-induced signals, since their mRNA levels were altered significantly by *Myostatin* suppression (Fig. 6). It is likely lack of available antibodies or these proteins are expressed too low to be detectable in the sheep myoblasts. Combined, we presently can confirm that signals of Ezh2, Akt, mTOR, p38MAPK, and Erk1/2 are involved in regulating the myoblast proliferation.

In addition, there were differences in the consistence of the expression patterns at the mRNA and protein levels for the genes examined. The mRNA profiles of *Ezh2*, *CyclinE* and *mTOR* were consistent with their protein profiles in GM, whereas the expression patterns of *Akt*, *p38MAPK*, *Cdk2*, and *Erk1/2* were not identical between the mRNA and the protein. (Figs. 4 and 5). This suggests that gene expression is probably modulated by *Myostatin* through various mechanisms.

### Src, Ezh2, Akt, mTOR, p38MAPK, and Erk1/2 involve in inhibition of myoblast differentiation by Myostatin knockdown

**Fig 7:**
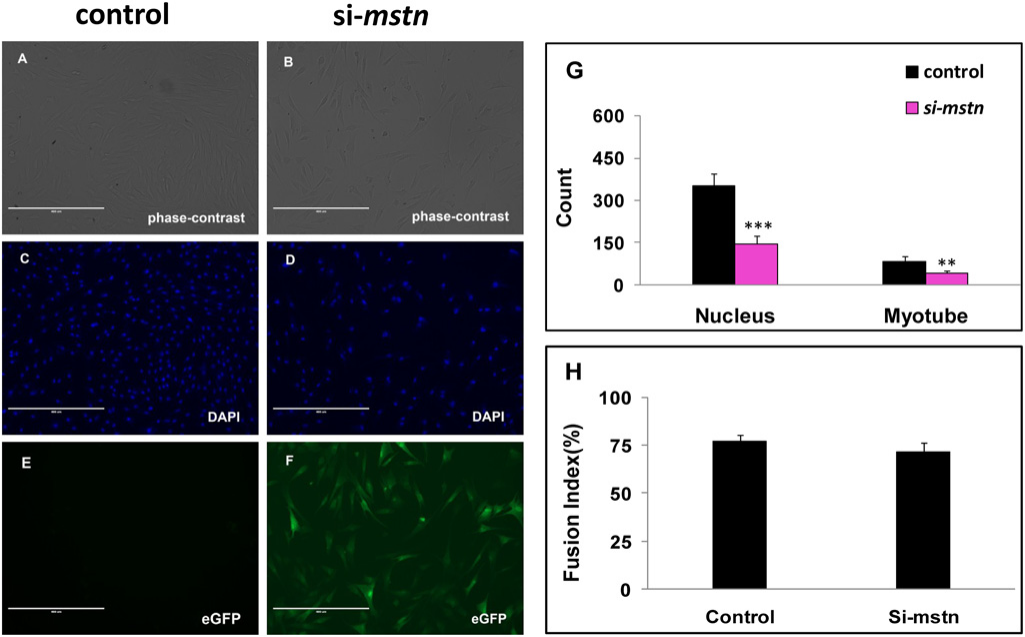
*Mstn* knockdown suppresses the sheep myoblast differentiation. Sheep myoblasts harboring *Myostatin* shRNAs and the negative control were cultured 3 days post-infection in DM. (A, B): phase contract photos; (C, D): nuclei stained by DAPI; (E, F): those cells harboring *Myostatin* shRNAs express eGFP; (G): counts of myotube and DAPI stained nucleus in a given area of view. (H): the fusion index of sheep myoblasts. Results are presented as the mean + SD of five replicates at least. The position of the samples on the plate was randomly assigned. ** P < 0.01, *** P < 0.001.

When the sheep myoblasts were cultured in differentiation medium (DM) for 3 days, *Myostatin* knockdown significantly decreased the cell and myotube counts (P<0.005) and delayed slightly the myogenic differentiation by reducing the fusion index (P>0.05), as compared with the control (Fig. 6). qPCR analysis demonstrated that inhibition of *Myostatin* significantly changed the transcription of *Catenin*, *Sfrp1*, *Hbp1*, *Erk1/2*, *Src*, *Histone h3*, *Smad3*, *Rb*, *Cdk2*, *Ezh2*, *p21*, and *Pax7* (Figs. 4 and 6). At the protein level, *Myostatin* suppression significantly increased expression of Cdk2, CyclinE, p-Erk1/2, p-Akt, p-mTOR, p38MAPK, Src, p-Src, and p-Ezh2 in the sheep myoblasts (Fig. 5). Similar to the result in GM (Fig. 6), Catenin, Sfrp1, Hbp1, Histone h3, Smad3, Rb, and p21 were still not detectable by Western blot (data were not shown) in DM. However, they probably play a role in regulation of myoblast differentiation. Here, higher expression of Cdk2 and CyclinE in the treatment indicate that more infected myoblasts are induced into apoptosis, thus resulting in the marked reduction of cell and myotube counts in DM (Fig. 7). Unlike the gene expression profiles of mRNA and protein in GM, expression of *Src* was upregulated by *Myostatin* suppression in DM, whereas the protein profiles of *Ezh2*, *Akt*, *p38MAPK*, *mTOR*, and *Erk1/2* were not consistent with their mRNA profiles by *Myostatin* inhibition in DM. Generally, these results clearly illustrate that signals of Src, Ezh2, Akt, mTOR, p38MAPK, and Erk1/2 are associated with the *Myostatin*-regulated myoblast differentiation.

### Inhibition of *Src*, *Ezh2*, and *Akt* by siRNA suppress *Pax7* expression in the sheep primary myoblasts

**Fig 8:**
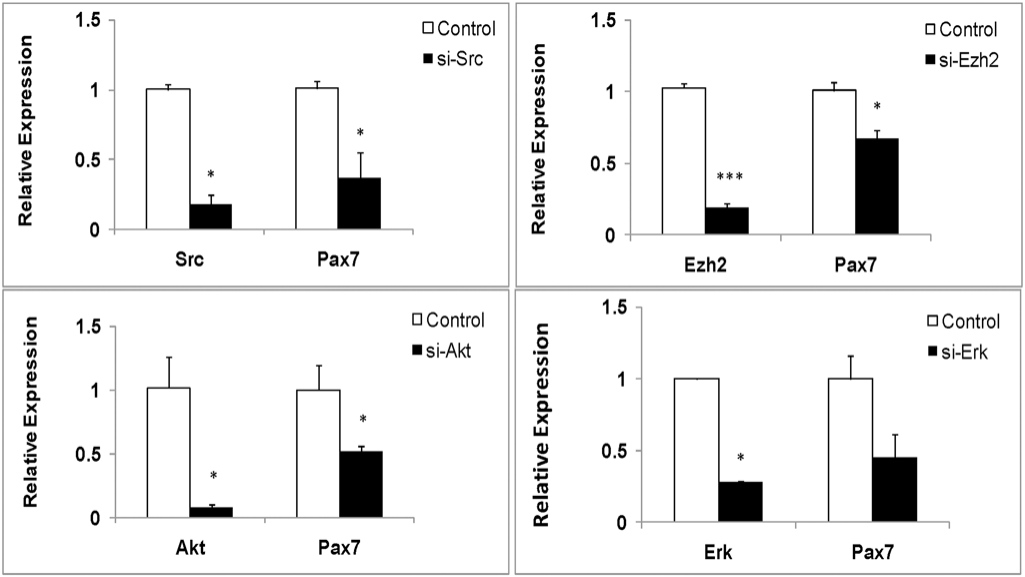
*Pax7* expression is affected by *Src*, *Ezh2*, and *Akt* suppression by siRNA in the sheep primary myoblasts. The sheep primary myoblasts were transfected with *Akt*-siRNA, *Ezh2*-siRNA, *Src*-siRNA, and the controls in serum-free medium using Lipofectamine 2000. After cultured 3 days in GM, cell lysates were used for gene expression analysis by qPCR in each group. For qPCR analysis, gene expression was quantified relative to *GADPH* expression using the 2-△△Ct method. The expression data in every group are shown as the mean + SD (n = 3) * P < 0.05, *** P < 0.001.

To further validate the downstream events of Myostatin-pax7 pathways (Fig. 1), we isolated the primary myoblasts from the muscle of newborn lamb to examine the *Pax7* expression. At the same time, we chemically synthesized the siRNAs for inhibition of *Src*, *Ezh2*, and *Akt* in the primary myoblasts, respectively. qPCR analysis illustrated that the *Pax7* mRNA level was significantly downregulated by either of the siRNAs for *Src*, *Ezh2*, and *Akt* in the primary myoblasts, compared with their controls (Fig. 8). These findings indicate that Pax7 is the downstream target of Src, Ezh2, and Akt signals.

In general, the present data in this study confirmed the existence of the Myostatin- Akt/Src/Ezh2-Pax7 pathways in muscle, which probably affect the behaviors of the myogenic cells at early myogenic stage.

## Discussion

Although the function of *Myostatin* for control of skeletal muscle mass is well documented, the underlying mechanism for this action of *Myostatin* is still not fully understood. It has been established that *Myostatin* negatively regulates proliferation of various myogenic cells [38, 40, 41]. However, the role of *Myostatin* in differentiation of myogenic cells is still controversial and in debate. Some demonstrate the inhibitory effect of Myostatin on myoblast differentiation in various species [23, 42–45], whereas others report the stimulatory effect of Myostatin on differentiation of myogenic cells [46–49]. Interestingly, Myostatin exerts an inhibitory effect on differentiation of adult-like myogenic cells such as C2C12 myoblasts and human myoblasts, while those myogenic cells whose differentiation was promoted by Myostatin are embryonic/fetal myoblasts, muscle progenitor cells (Pax3 and/or Pax7 positive). There are differences in cellular identities between the two classes of cell lines. Our present study indicates Myostatin inhibits the proliferation (Fig. 3) and stimulates the differentiation of the sheep myoblasts (Fig. 7). The lower cell counts in the sh*MSTN*-affected groups in DM suggest that the apoptosis occurred at that time. The significance of Cdk2 and CyclinE for apoptosis is complicated by the fact that the model cells are either proliferating [50] or undergoing apoptosis as a consequence of growth factor withdrawal [51]. In the present study, upregulation of both CyclinE and Cdk2 levels (Fig. 5) suggest that the sheep myoblasts enter a proliferative process in GM, whereas part of myoblasts enter the apoptotic process in DM in response to *Myostatin* suppression. However, a recent study in the sheep myoblasts shows a inhibitory effect on differentiation [43], which is opposite to ours. The reason why there are differences between these two studies remain to be elucidated. Nevertheless, it is established that downregulation of Pax7 is required for myogenic progression, and upregulation of Pax7 expression inhibits myogenic cell differentiation [13–15]. Moreover, a recent study demonstrates that a genetic inactivation of *Myostatin* blocks the myotube formation in vitro, but the myogenic capacity is recovered in vivo under the influence of the Myostatin+ host-tissue environment in mice [49]. This illustrates that Myostatin expression is indispensable for myogenic differentiation. Thus, in the developmental context, endogenous Myostatin promotes myogenic differentiation by fine-tuning the proliferation and differentiation of myogenic cells. Furthermore, evidences suggest that Myostatin acts in an autocrine manner affecting the behaviors of myogenic cells [52, 53]. Therefore, it is not surprising that there is a diversity of myogenic effects between exogenous and endogenous Myostatin. The effect of Myostatin on myogenic cells depends not only on the cellular identity but also on the external environment where they live.

Up to date, only the Erk1/2 signal is reported to be associated with Myostatin-modulated Pax7 expression in adult mice muscle [34]. In the present study, we firstly show that at least signals of Src, Ezh2, and Akt besides Erk1/2, are involved in Myostatin-Pax7 pathways. Especially, the present results (Fig. 3) well explain our previous observation that higher Pax7 expression of muscle in Texel enables the higher myogenic potential, compared with its wild type counterpart [22].

*Src* (v-src avian sarcoma (schmidt-ruppin A-2) viral oncogene homolog), not only is a protein-coding gene, but also is affiliated with the lncRNA class. The proto-oncogene *v-src* can promote the myoblast proliferation [54], but inhibits its differentiation [55] mainly through direct regulation of MyoD family [56]. However, inhibition of *Src* suppresses the formation of myotubes and expression of myogenin [57]. These reflect the diverse effects on myogenic outcome between the endogenous Src and exogenous v-Src. Our findings demonstrated that the Myostatin-affected upregulation of endogenous Src suppressed the myogenic differentiation (Figs. 5 and 7), while inhibition of *Src* suppressed *Pax7* mRNA expression in the sheep myoblasts (Fig. 8), which is consistent with the roles of Pax7 in myogenesis [13–15]. Unlike the results of myoblast proliferation in the present study, we revealed that Src is involved in the regulation of the sheep myoblast differentiation except Akt and Ezh2. Recent findings show that Pax7 repression in embryonic stem cells is involved in p38alpha signaling during muscle satellite cell differentiation [58, 59], which provides a well-established evidence of a regulatory relationship between Pax7 and p38 signal in differentiation. Interestingly, Src also acts as an upstream regulator on activation of MAPKs in skeletal muscle cells [60–62]. This suggests that the feedback loops may exist between Src and the canonical signals such as Erk1/2, Akt, and p38 in muscle cells.

Ezh2 (enhancer of zeste homolog 2), a histone methyltransferase and catalytic component of polycomb repressive complex 2 (PRC2), epigenetically regulates chromatin structure, gene expression, and recruitment of DNA methyltransferases for gene silencing in embryonic and adult stem cells [26, 63]. Studies show that Ezh2 is required for appropriate proliferation of the satellite cells (Pax7+myogenic cells) and postnatal muscle growth in mice [64]. The inhibitory effect of Ezh2 on the differentiation is also observed in C2C12 myoblasts [65], which is consistent with our results (Fig. 5). In addition, Cdk1-mediated phosphorylation of Ezh2 is reported to be important for cell proliferation [66]. A recent study demonstrates that TNF-activated p38alpha kinase promotes the phosphorylation of Ezh2, leading to the repression of Pax7 expression in satellite cells [59]. In the present study, we firstly demonstrated the effects of Myostatin on Ezh2 and Pax7 in the proliferating and differentiating sheep myoblasts (Figs. 4 and 7), and confirmed the existence of a Myostatin-Ezh2-Pax7 pathway in the sheep myoblasts. Although Pax7 protein was undetectable in the sheep myoblasts by Western blot in the present study (data not shown), the great variations in *Pax7* mRNA levels in muscle and myoblast (Figs. 2 and 4) substantially changed expression of its downstream genes, such as *Myf5* and *MyoD*, which subsequently affected the sheep myoblast proliferation and differentiation. However, it is still a challenge to obtain the pure myogenic progenitor cell lines such as the embryonic or fetal myoblasts, and the satellite cells in vitro because there are lack of the efficient techniques so far.

In addition, the observation that variations in expression of the signals examined at mRNA and protein levels under different conditions (GM or DM) (Figs. 4 and 5) suggests the complexity of signaling transduction, namely the physiological effects of a signal on cells depend not only on expression of the signal itself but also on the environment where cells live. The present study extends the regulatory network of Myostatin-Pax7 pathways and further illustrates that Myostatin as a global regulator participates in the epigenetic events involved in myogenesis.

## Conclusion

Myostatin is required for normal myogenic differentiation of the sheep myoblasts. Myostatin signals to Pax7 at least through Ezh2, Src, and Akt in the sheep myoblasts. In addition, other signals such as Erk1/2, p38MAPK, mTOR, Wnt, Bmp2, Smad, Tgfb1, and p21 are most probably involved in the myostatin-affected proliferation and differentiation.

## Supporting Information

S1 Table List of differentially expressed genes in Longissimus muscle across the five fetal stages in Texel sheep. (XLS)

S2 Table List of differentially expressed genes in Longissimus muscle across the five fetal stages in Ujumqin sheep. (XLS)

S1 File The primary antibodies for Western blot analysis. (DOC)
