# Supplementary material for: Signals of Ezh2, Src, and Akt Involve in Myostatin-Pax7 Pathways Regulating the Myogenic Fate Determination during the Sheep Myoblast Proliferation and Differentiation
Source: PLoS One. 2015 Mar 26;10(3):e0120956. doi: 10.1371/journal.pone.0120956 (PMC4374906; doi:10.1371/journal.pone.0120956)
Supplement: S1 File — (DOC) [file pone.0120956.s003.doc]

The primary antibody and its dilution ratio for Western blot in the present study is indicated as following:

rabbit monoclonal anti-ERK1 (pY204) + ERK2 (pY187) antibody [EP197Y] (ab76299, Abcan), 1:1000; rabbit-polyclonal anti-Cyclin E antibody (ab88259, Abcam), 1:1000; rabbit polyclonal anti-Src (phospho Y529) antibody [Y232] (ab32078, Abcam), 1:1000; mouse monoclonal anti-Src antibody [Clone 327] (ab16885, Abcam), 1:1000; rabbit monoclonal anti-KMT6/Ezh2 (phospho T487) antibody [EPR1410] (ab109398, Abcam) 1:500; rabbit polyclonal anti-Kmt6 / Ezh2 antibody (ab124522, Abcam), 1:125; mouse monoclonal anti-Akt1 (phospho T308) antibody [18F3.H11] (ab105731, Abcam), 1:1000; rabbit polyclonal anti-Cdk2 (phospho T39) antibody (ab79379, Abcam), 1:100; rabbit monoclonal anti-p21 (phospho S146) antibody [EP1138] (ab92675, Abcam), 1:100; rabbit polyclonal anti-Smad3 (phospho S423 + S425) antibody (ab51451, Abcam), 1:250; rabbit polyclonal anti-Histone H3 (tri methyl K4) antibody - ChIP Grade (ab8580, Abcam), 1:50; rabbit polyclonal anti-Histone H3 (phospho S10) antibody - Mitosis Marker (ab5176, Abcam), 1:100; rabbit polyclonal anti-Bmp2 antibody [65529.111] (ab6285, Abcam), 1:100; rabbit polyclonal anti-Myosin Light Chain 2 Antibody (3672, Cst), 1:500; rabbit polyclonal anti-mTOR (phospho S2448) antibody (ab84400, Abcam), 1:1000; rabbit polyclonal anti-Gdf8 / myostatin antibody (ab98337, Abcam), 1:50; rabbit monoclonal anti-Phospho-p38 MAPK (Thr180/Tyr182) (12F8) Antibody(4631S, Cst), 1:250; rabbit polyclonal anti-Phospho-Rb (Ser807/811) Antibody(9308, Cst), 1:250; mouse monoclonal anti-Bmi1 antibody [1.T.21] - ChIP Grade (ab14389, Abcam), 1:100; rabbit polyclonal anti-Jnk1 (phospho T183 + Y185) antibody (ab18680, Abcam), 1:250.
